# Advances in biosynthesis of scopoletin

**DOI:** 10.1186/s12934-022-01865-7

**Published:** 2022-08-02

**Authors:** Bo-Tao He, Zhi-Hua Liu, Bing-Zhi Li, Ying-Jin Yuan

**Affiliations:** Frontiers Science Center for Synthetic Biology and Key Laboratory of Systems Bioengineering (Ministry of Education), School of Chemical Engineering and Technology, Tianjin University, Tianjin, 300072 People’s Republic of China

**Keywords:** Scopoletin, Coumarins, Synthetic biology, Biosynthesis, Microbial cell factory

## Abstract

Scopoletin is a typical example of coumarins, which can be produced in plants. Scopoletin acts as a precursor for pharmaceutical and health care products, and also possesses promising biological properties, including antibacterial, anti-tubercular, anti-hypertensive, anti-inflammatory, anti-diabetic, and anti-hyperuricemic activity. Despite the potential benefits, the production of scopoletin using traditional extraction processes from plants is unsatisfactory. In recent years, synthetic biology has developed rapidly and enabled the effective construction of microbial cell factories for production of high value-added chemicals. Herein, this review summarizes the progress of scopoletin biosynthesis in artificial microbial cell factories. The two main pathways of scopoletin biosynthesis are summarized firstly. Then, synthetic microbial cell factories are reviewed as an attractive improvement strategy for biosynthesis. Emerging techniques in synthetic biology and metabolic engineering are introduced as innovative tools for the efficient synthesis of scopoletin. This review showcases the potential of biosynthesis of scopoletin in artificial microbial cell factories.

## Introduction

Coumarins are a family of secondary metabolites that are widely found in various plants, fungi, and microorganisms. According to their biosynthetic pathways, coumarins are divided into three main groups, i.e., the shikimate acid pathway, cinnamic acid pathway, and phenylalanine metabolic pathway [1]. Coumarins are organic heterocyclic compounds derived from the phenylpropane. The parent nucleus skeleton structure is benzo-α-pyrone, which can be divided into four categories according to the different substituents on the benzene ring. These are known as simple coumarins, furan coumarins, pyran coumarins, and other substituted coumarins [2]. There are a large number of descriptions in existence of the phenylpropanoid biosynthetic pathway for several classes of compounds, such as flavonoids and lignins. Nevertheless, few studies have been conducted on the in-depth prospects of biosynthesis routes required for coumarins [3].

**Fig. 1:**
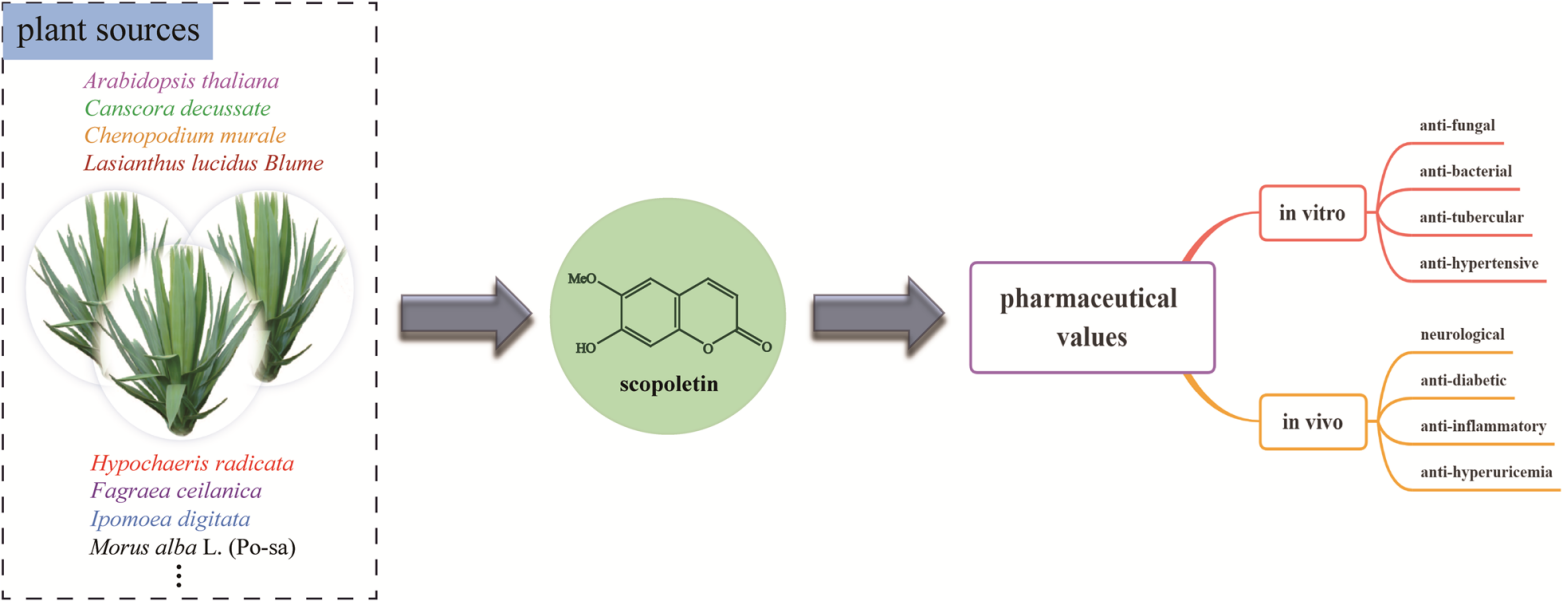
The plant sources and pharmaceutical values of scopoletin

Scopoletin (7-hydroxy-6-methoxy coumarin), is a typical representative of the coumarin family. The substance is derived from the carbon skeleton C6-C3 and contains a flavonoid skeleton core in a 1,2-benzopyrone structure [4], with methoxylation and hydroxylation modifications of the benzene ring. As shown in Fig. 1, it is a phenolic coumarin compound found in *Arabidopsis thaliana* [5]and other plants [6–12] with the phenylpropane pathway. Its medicinal value has been of great interest around the world [13]. Scopoletin (SPT) has been reported in vitro pharmacological activity, including anti-bacterial [6], anti-fungal [14, 15], anti-tubercular [7], and anti-hypertensive [16] properties. The proven in vivo pharmacological activity includes anti-inflammatory [17–21], neurological [4, 22–24], anti-diabetic [20, 25] and anti-hyperuricemia [26] properties. Fraxetin (7,8-dihydroxy-6-methoxy coumarin) is synthesized from scopoletin by the hydroxylation at the C8 position and is involved in iron metabolism in plants [27, 28]. Fraxetin was also found to exert beneficial effects including anti-inflammatory [29], anti-hyperglycemic [30], antitumor [31, 32], etc. Sideretin (5,7,8-trihydroxy-6-methoxycoumarin) can efficiently mobilize and reduce insoluble Fe^3+^, and rescue the chlorotic phenotypes of wild-type plants under conditions of deficient iron availability [33].

Currently, coumarins are mainly produced by chemical synthesis, and extraction from plants. With the increasing demand for coumarins, the traditional method of extraction from plants or chemical synthesis is considered unsustainable, due to the high production costs involved. Recently, artificial microbial cell factories seems to be a promising strategy for the large-scale and economically viable production of coumarins. With this aim in mind, this review focuses on the production of scopoletin in microbial cell factories (MCFs) using synthetic biology methods.

## Biosynthesis pathway of scopoletin

The metabolic pathway for scopoletin biosynthesis involves multiple types of chemical reactions catalyzed by the following catalytic enzymes: tyrosine ammonia lyase (TAL), phenylalanine ammonia lyase (PAL), 4-coumarate CoA ligase (4CL), feruloyl-CoA synthase (FCS), cinnamate-4-hydroxylase (C4H), 4-hydroxyphenylacetic acid 3-hydroxylase A (HHA), coumarate-3-hydroxylase (C3H), feruloyl-CoA 6'-hydroxylase (F6’H), coumaroyl CoA 2'-hydroxylase (C2'H), coumarin synthase (COSY), scopoletin 8-hydroxylase (S8H), caffeoyl-CoA *O*-methyltransferase (CCoAOMT), and the cytochrome P450 family B2 subfamily C polypeptide 4 enzyme (CYP82C4).

**Fig. 2:**
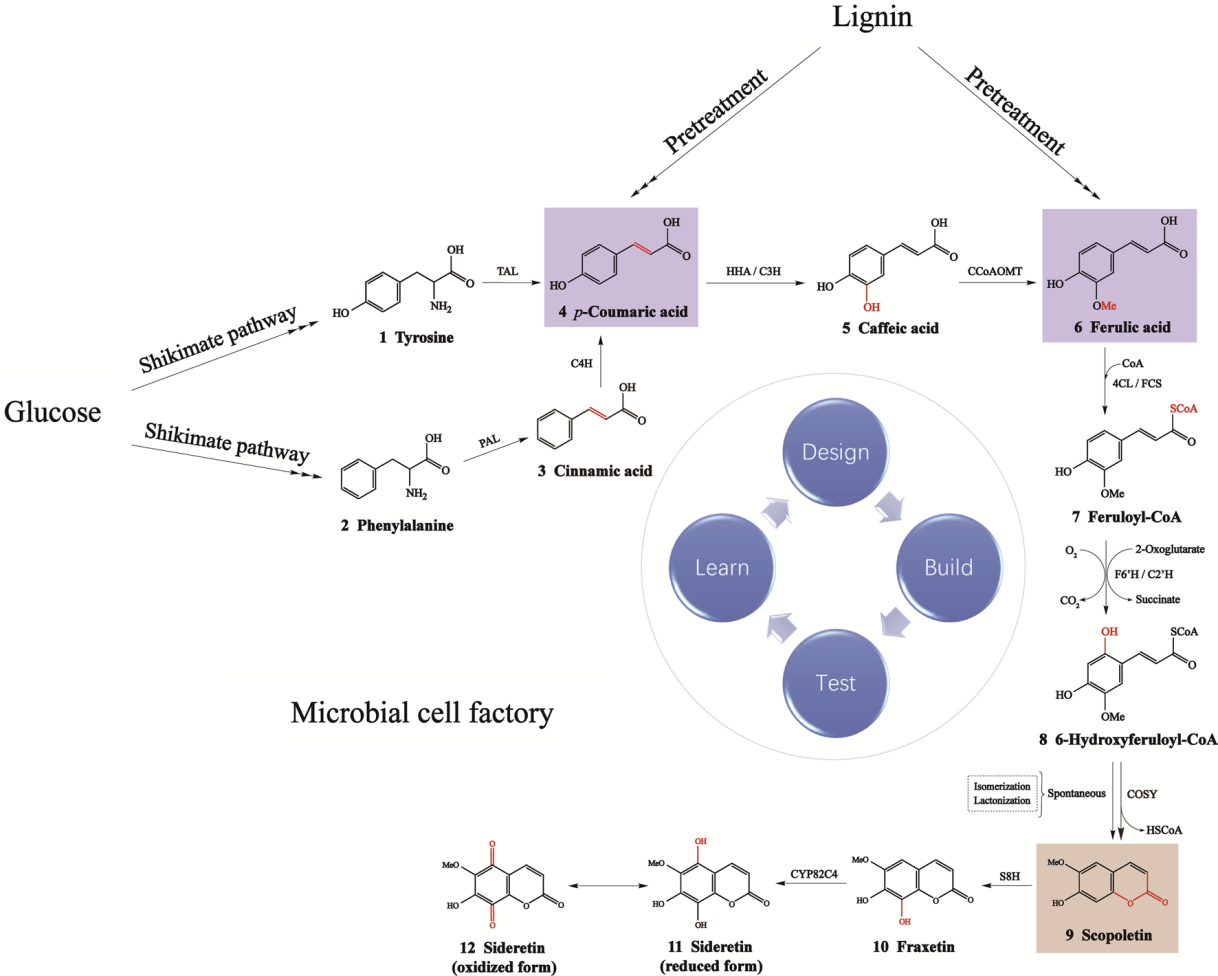
Biosynthetic pathway of scopoletin from glucose or lignin-derived aromatics. The compounds in the purple box are the two major monomers derived from lignin after pretreatment. The compound in the orange box is scopoletin, which is the target product produced by the microbial cell factory in this paper. TAL: tyrosine ammonia lyase; PAL: phenylalanine ammonia lyase; 4CL: 4-coumarate CoA ligase; FCS: feruloyl-CoA synthase; C4H: cinnamate-4-hydroxylase; HHA: 4-hydroxyphenylacetic acid 3-hydroxylase A; C3H: coumarate-3-hydroxylase; F6’H: feruloyl-CoA 6'-hydroxylase; C2'H: coumaroyl CoA 2'-hydroxylase; COSY: coumarin synthase; S8H: scopoletin 8-hydroxylase; CCoAOMT: caffeoyl-CoA *O-*methyltransferase; CYP82C4: cytochrome P450 family B2 subfamily C polypeptide 4 enzyme. Three consecutive arrows indicate multiple biosynthetic steps

Scopoletin and other coumarin derivatives (fraxetin and sideretin) can be produced in microbial cell factories from both glucose and lignin-derived aromatics (Fig. 2). When glucose is used as substrate [34], tyrosine and phenylalanine are generated via the shikimate acid pathway. PAL catalyzes phenylalanine to generate cinnamic acid and then yield *p*-coumaric acid via C4H catalysis. Tyrosine is catalyzed by TAL to generate *p*-coumaric acid directly. With the *o*-hydroxylation of *p*-coumaric acid by HHA or C3H, caffeic acid is produced and can be further methylated via CCoAOMT to generate ferulic acid. The formation of feruloyl CoA is catalyzed by 4CL or FCS. F6'H or C2'H is capable of catalyzing *o-*hydroxylation of feruloyl CoA and subsequent spontaneous reactions (isomerization and lactonization), leading to the formation of scopoletin. Recently, coumarin synthase (COSY) was reported to further enhance the formation of scopoletin. COSY is an acyltransferase catalyzing the trans–cis isomerization and lactonization in coumarin biosynthesis in plants [35]. COSY was found to be a key enzyme in coumarin biosynthesis, providing a route for increasing coumarin production in crops or microbes [35]. It was demonstrated that COSY significantly increased the reaction efficiency from 6-hydroxyferuloyl-CoA into scopoletin [35]. Scopoletin 8-hydroxylase (S8H) has been reported as a strong Fe-responsive gene encoding a 2-oxoglutarate-dependent dioxygenase [27, 28], and it can also catalyze scopoletin to form fraxetin in plants via *o*-hydroxylation at the C8 position. Overexpression of S8H improved fraxetin biosynthesis in *Escherichia coli* [36]. In the presence of CYP82C4, fraxetin can be converted to the oxidized and reduced forms of sideretin [33].

The synthesis of coumarins can also be produced from aromatic compounds derived from lignin by pretreatment [37, 38]. For example, two major monomers, *p*-coumaric acid and ferulic acid, can be generated in alkaline hydrolysate from pretreatment and used for the biosynthesis of scopoletin. By means of the metabolic funnel principle, the same steps described above catalyze these compounds to produce scopoletin [39]. As one of the three major components in lignocellulose, and the most abundant renewable aromatic resource on earth, lignin could be a promising feedstock for production of aromatic derivatives, because of the significant shortening of the metabolic pathways.

## Regulation of scopoletin biosynthesis

**Fig. 3:**
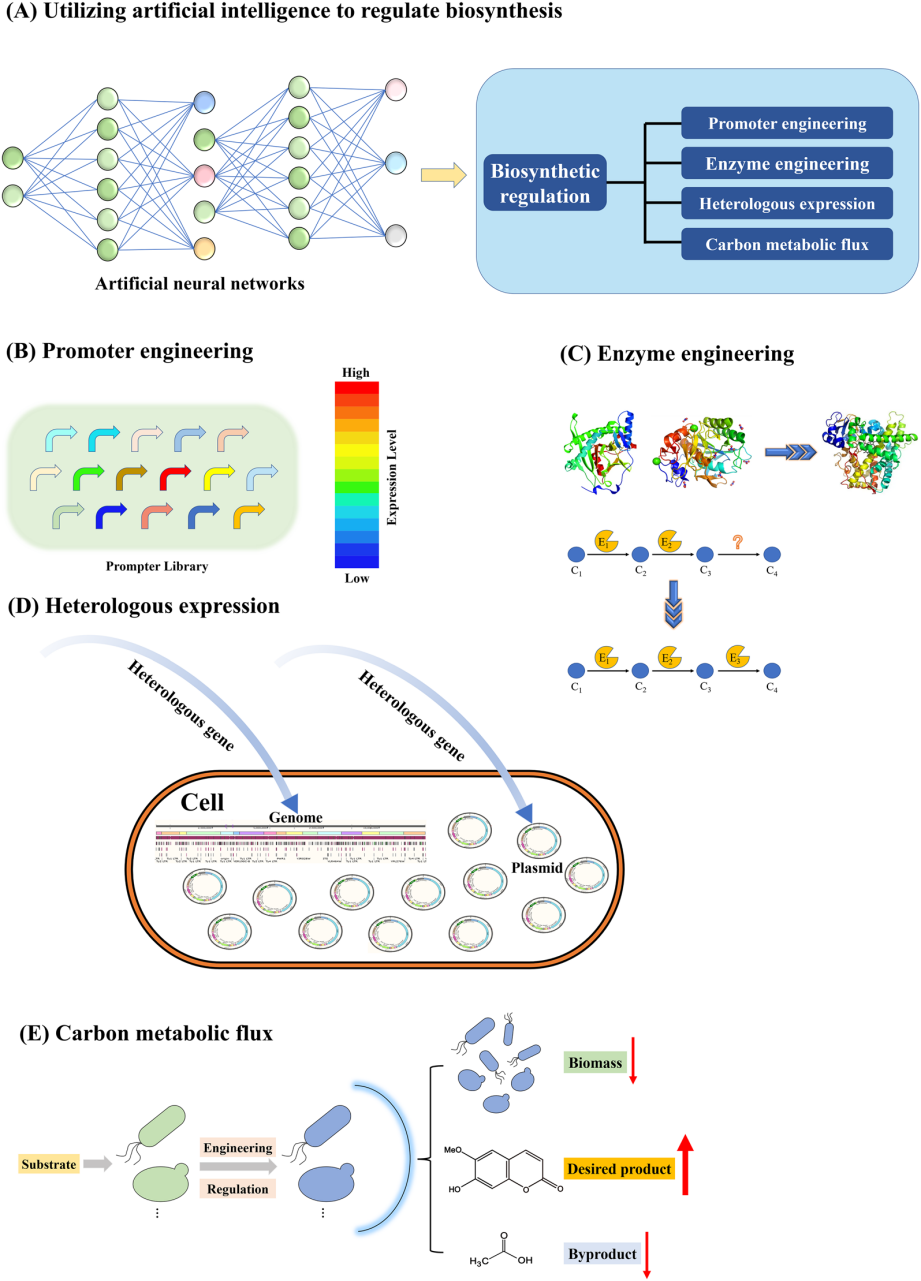
Synthetic biology methods for biosynthetic regulation to increase the titer, rate, and yield (TRY) of product. **A** Diagram of artificial neural network-guided biosynthetic regulation. **B** Pathway optimization based on the promoter library containing different expression intensities. **C** Novel enzyme search and initial enzyme modification, optimization and protein design. C_1_, compound 1; C_2_, compound 2; C_3_, compound 3; C_4_, compound 4; E_1_, enzyme 1; E_2_, enzyme 2; E_3_, enzyme 3. **D** The process for transforming exogenous genes and exogenous pathways into engineered strains by means of plasmid construction or genomic integration. **E** The engineering process reduces biomass, reduces the loss of carbon sources in branching pathways, reduces metabolic competition between desired products and byproducts, and ensures the continued accumulation of the desired products

In recent years, the biosynthesis of scopoletin has been developed in microbial cell factories, and a variety of regulation methods have been applied to improve scopoletin biosynthesis including promoter engineering, enzyme engineering, heterologous expression, and carbon metabolic flux (Fig. 3).

### Promoter engineering

Promoter engineering is a significant basis for regulating gene expression and optimizing metabolite biosynthesis in metabolic engineering and synthetic biology. Gene expression levels must be coordinated with changes in intracellular and extracellular conditions to maintain the homeostasis of the metabolic network. Therefore, the dynamic regulation of gene expression requires more flexible regulatory promoters [40]. Around 100 natural promoter 5’-UTR complexes from *E. coli* were screened and identified based on RNA-seq data for accurate analysis of the transcriptional network in *E. coli* cells [41]. A de novo synthetic promoter library was applied in *Bacillus subtilis* for the production of inosine and acetaldehyde, and their yields increased sevenfold and 1.4-fold, respectively [42]. Promoter libraries was also used to optimize gene expression to improve the production of (2S)-naringenin accumulation [43]. Dozens of promoters were amplified from *Saccharomyces cerevisiae* genome to construct a promoter library with gradient intensities, and high-throughput screening techniques were used to screen the strains with an enhanced capacity for (2S)-naringenin production. Finally, 1.21 g/L (2S)-naringenin was achieved at the 5-L fermenter, which represented the highest naringin yield. Based on promoter optimization and directed evolutionary strategies, (2S)-eriodictyol from (2S)-naringenin was efficiently synthesized in *S. cerevisiae* [44]. A random assembly library was obtained through promoter engineering, and the accumulation efficiency of (2S)-eriodictyol of strains in the random library was screened in high throughput. A final titer of (2S)-eriodictyol reached 3.28 g/L in a 5-L fermenter. Upregulation or downregulation of gene expression by changing the promoters can alter metabolic flux to improve the production of target metabolites [45]. Recently, the prediction of expression strength of the *S. cerevisiae* promoter was achieved through a combination of computer simulation and wet experimental verification, showing the potential to improve the biosynthesis of natural products such as coumarins [46]. Inducible promoter engineering has become a promising strategy to improve metabolite production. When the constitutive promoter TPI1 of the *4CL* gene was changed to galactose-inducible promoter Gal1 via CRISPR-Cas9, an increased yield of the target product was achieved [37]. As coumarin biosynthetic pathways are composed with dozens of enzymes, promoter engineering would be very efficient approach to regulate the pathway. According to previous study, inducible promoter engineering, such as application of end-product inducible promoter, may be helpful to improve coumarin production.

### Enzyme engineering

Enzyme engineering is also important to improve the yield of natural products. CCoAOMT is a key enzyme for the production of feruloyl CoA by catalyzing caffeic acid, and it is also involved in the biosynthesis of lignins and scopoletin in *Arabidopsis* roots [47]. Previous studies have confirmed that the accumulation of coumarins is hindered with the loss-of-function of CCoAOMT in mutants [48]. F6'H [47] or C2'H [49] is involved in the biosynthesis of scopoletin, since feruloyl CoA is modified with the hydroxylation at position 6 by these enzymes. The *Arabidopsis* mutation *f6'h1* can successfully produce coumarin derivatives, such as scopoletin, fraxetin and sideretin [27, 33, 47, 50, 51].

Methylation and hydroxylation are the two important reactions in scopoletin biosynthesis. The yields of coumarins are generally low, which is due to the low activity of the methyltransferases and hydroxylase to a great extent. Modification of methyltransferases by rational protein engineering enhanced their binding capacity with substrates, and activity of *O*-methylation of *N*-acetylserotonin (NAS) was improved by 9.5 fold [52]. In terms of hydroxylation, the development of a highly active PobA, a *p*-hydroxybenzoate hydroxylase from *Pseudomonas aeruginosa*, variant for hydroxylating 3,4-dihydroxybenzoic acid (3,4-DHBA) into gallic acid (GA) via structure-based protein engineering approach was reported [53]. Multiple mutations by means of molecular dynamics simulations and binding free energy analysis can be designed to strengthen the interactions between the substrate binding pocket of the enzyme. Overall, enzyme engineering will be a promising strategy for exploiting the methylase and hydroxylase with high substrate activity.

Many factors, such as substrate specificity and enzymatic activity of key enzymes, affect conversion and production efficiency. Nowadays, artificial intelligence and automated platforms, such as bioinformatics and machine learning, help in the engineering and optimization of enzymes. The mining new enzymes can be accelerated with the help of a computational method called AlphaFold 2 [54]. This emerging technology can be used to predict protein structures with atomic-level accuracy without requiring prior knowledge of their structures, which in turn can guide enzyme design In terms of modification and optimization of the original enzymes, characterization of more than 1000 enzyme mutants was achieved in a single experimental test by establishing a new system platform for high-throughput microfluidic enzyme kinetics (HT-MEK) [55]. In the synthetic pathway of scopoletin, 4CL has the potential to catalyze *p*-coumaric acid and caffeic acid as well as ferulic acid due to the weak substrate specificity, and the specific substrate affinity can be increased by engineering of 4CL. In the cell factories for the production of umbelliferone, virtual screening based on the binding energies was used for the selection of 4CL, and the site-specific mutagenesis-based protein engineering approach indicated that double mutation of Q272H and F267L may improve the yield of umbelliferone [56]. Therefore, enzyme engineering plays an important role in modifying and optimizing key catalytic properties of enzymes, and shows significant promise in regulating the biosynthesis pathway of the coumarin derivatives.

### Regulation of heterologous expression

Heterologous expression is fundamental in the production of natural products in microbial cell factories. In order to improve the production, the strategies about heterologous expression include overexpression of key genes, fusion expression of sequential enzyme genes, expression of key with specific locations and so on. With the co-expression of 4CL from *Petroselinum crispum* and F6'H1 from *Arabidopsis thaliana*, the engineered strain achieved 27.8 mg/L scopoletin using ferulic acid as substrate [57]. By fusing F6'H1 from *Ipomoea batatas* with glutathione S-transferase (GTS) and expressing 4CL from *Oryza sativa* in *E. coli*, the yield of scopoletin increased to 79.5 mg/L using ferulic acid as a substrate [34]. Ferulic acid was transformed into scopoletin in *S. cerevisiae* using the heterologous expression [38]. Fusion expression of feruloyl coenzyme A 6'-hydroxylase (F6'H1) from *Arabidopsis thaliana* and 4-coumarate coenzyme A ligase (4CL) from *Petroselinum crispum* significant improved the production of scopoletin in *S. cerevisiae*. Experimental screening was conducted with a devise of different lengths, and forward and reverse linkers. Different linkers between the two fusion expressed genes also affect the conversion. The linker of (Gly-Gly-Gly-Gly-Gly-Gly-Ser)_4_ showed a 3.3-fold increase in scopoletin production and a final titer of 3.42 mg/L.

Organelle localization of enzymes are also important to improve the product yield. Enzymes can be localized in different organelles, including the endoplasmic reticulum (ER), lipid body (LB) and peroxisome, and so on. Compartmentalization in cellular organelles can isolate synthetic pathways from competing pathways, and provide a suitable environment for biosynthesis. Synthetic pathways targeting to peroxisomes increased the production of fatty-acid-derived fatty alcohols, alkanes and olefins [58]. Condensation of Multienzymes in *E. coli* compartmentalized the cytosolic space into regions of high and low enzyme density and led to a significant enhancement of α-farnesene production [59]. The localization of different lipases into the organelles of lipolytic yeast was proposed to improve triglyceride derivative production by compartmentalization [60]. Due to the specific cofactor requirements and product activities, it is expected that the yield of coumarins can be improved by organelle localization in the future.

### Regulation of carbon metabolic flux

Carbon metabolic flux regulation is important for improving the efficiency of microbial cell factories for natural products. Carbon metabolic flux can be regulated by the inhibition of side reactions, genomic disturbance and screening, and so on. The metabolic pathways of coumarin synthesis are usually long pathways with dozens of reactions. The side reactions would reduce the metabolic flux to the target products. It is crucial to reduce metabolic competition between target products and byproducts [58, 61–63]. In the cell factory for umbelliferone biosynthesis, both prephenate dehydratase (*pheA*) and *o-*aminobenzoic acid synthase (*trpE*) catalyze the side reactions, and they are knocked out to improve carbon flux of tyrosine, which is the precursor for umbelliferone biosynthesis [56].

Furthermore, gene editing, gene circuits [64], and other biotechnologies can be employed to assemble and optimize multiple biological components, creating biological bricks, and thus improving the biosynthesis efficiency of target products. Commonly used gene editing methods include zinc-finger nucleases (ZFNs) [65], transcription activator-like effector nucleases (TALENs) [66], CRISPR [62, 67], FLP/FRT [63] and Cre/Loxp [66–68], etc. *E. coli* W3110 can only accumulate 5.54 g/L pyruvate, but accumulate large amounts of byproducts including lactate, acetate and formate. In order to improve the accumulation of pyruvate in the strain, the FLP/FRT gene editing technology was used to knock out the genes encoding lactate dehydrogenase (*ldhA*), pyruvate oxidase (*poxB*), pyruvate formate lyase (*pflB*), phosphotransacetylase (*pta*), and acetate kinase A (*ackA*), which led to accumulate 20.9 g/L pyruvate [63]. Based on Chromosome Rearrangement and Modification by LoxP-mediated Evolution (SCRaMbLE) system in diploid yeast strain, a strategy called Multiple SCRaMbLE Iterative Cycling (MusIC) was developed to increase the production of carotenoids up to 37.39 mg/L [69]. During the synthesis of adipic acid, the yield of adipic acid was reduced due to the accumulation of by-products such as lactic acid and butyric acid. The genes encoding l-lactate dehydrogenase (*ldhA*) and acetyl-CoA acetyltransferase (*atoB*) were deleted individually by CRISPR-Cas9, and the yield of adipic acid was increased from 49.5% to 61.7% and 68.5% theoretical yield in shaken flasks [62]. Gene circuits can help the distribution of carbon flux in the metabolic network and improve the synthesis of target products in engineered strains. After evaluation of the orthogonality and dynamic regulatory range of the quorum sensing (QS) systems, the QS system of *Vibrio fischeri* and *Enterococcus faecalis* was used to control the synthesis of medium-chain fatty acid pathway enzymes and the expression of the endonuclease MazF, ultimately altering the global distribution of cellular metabolic resources and the production of medium-chain fatty acid increased by 5.4-fold in a 5-L fermenter [70]. When CRISPR interference (CRISPRi) was used for the identification of potentially beneficial targets at the genome-wide level, 30 beneficial genes were identified from 108 targets associated with free fatty acids (FFA) metabolism, an additional 26 beneficial genes were also identified from those did not appear to be associated with FFA metabolism. According to these target genes, an engineered E. coli produced 30.0 g/L FFA in a fed-batch fermentation [71].

## Conclusions and future perspectives

Natural aromatic products, such as coumarins, are a group of chemicals of great industrial importance [72]. Within the coumarin family, hydroxycinnamic acids such as *p*-coumaric acid, caffeic acid, and ferulic acid are also commonly found in plants. With rapid developments in synthetic biology, the design and optimization of microbial cell factories has gradually opened up for natural aromatic product synthesis, applying a new approach for the synthesis of coumarins from renewable sources like lignin or its derived aromatics. Despite the efforts made, cytotoxicity induced by the accumulation of aromatic products remains a continuous challenge that cannot be ignored. Further optimization to improve the tolerance of microbial cell factories has become an essential step for industrial production. In addition, the tolerance of engineered strains can be enhanced by means of adaptive laboratory evolution and genomic rearrangement techniques, along with other methods for finding key tolerance elements and targets. Furthermore, the biosynthesis processes in microbial cell factories should be based on the concepts of economic benefit and low-carbon development. In conclusion, with developments in synthetic biology, the emergence and integration of more and more advanced technologies and genetic tools will accelerate the development of more efficient microbial cell factories and realize feasible industrial production for natural aromatic products.
